# Effect of Oxadiazolyl 3(2H)-Pyridazinone on the Larval Growth and Digestive Physiology of the Armyworm, *Pseudaletia separata*

**DOI:** 10.1673/031.008.1901

**Published:** 2008-03-12

**Authors:** Qingchun Huang, Yuping Kong, Manhui Liu, Jun Feng, Yang Liu

**Affiliations:** Shanghai Key Lab of Chemical Biology, School of Pharmacy, East China University of Science and Technology, Shanghai 200237, China

**Keywords:** oxadiazolyl 3(2H)-pyridazinone, toosendanin, *Pseudaletia separata*, process of growth, digestive physiology

## Abstract

The effect of oxadiazolyl 3(2H)-pyridazinone (ODP), a new insect growth regulator, on growth of larvae of the armyworm, *Pseudaletia separata* Walker (Lepidoptera: Noctuidae) was evaluated in comparison to the insecticide, toosendanin, a tetranortriterpenoid extracted from the bark of *Melia toosendan* that has multiple effects on insects. The digestive physiological properties of these compounds on insects were investigated by feeding them maize leaves dipped in these compounds. The results showed that ODP inhibited the growth of *P. separata* significantly, causing a slowed development and a prolonged larval period, smaller body size and sluggish behavior, delayed pupation and a reduced eclosion rate of pupae and adults. Moreover, ODP strongly inhibited the activities of weak alkaline trypsine-like enzyme, chymotrypsin-like enzyme and alpha amylase in the midguts of fifth instar *P. separata* larvae, *in vivo*, and inhibited the activity of alpha amylase, *in vitro.* These data suggest that ODP has severe consequences on the larval carbohydrate assimilation and/or nutrient intake and thereby causes inhibition of larval growth. The regulatory action of ODP on larval growth development was similar to that of toosendanin; both could be used to decrease the growth of insect populations.

## Introduction

Insect growth regulators control insect population, being known primarily as regulators of moulting, metamorphosis and many other physiological and developmental processes (Williams 1956; Fox 1990; Mondal et al. 2000). Non-steroidal dibenzoylhydrazines such as RH5849 and RH5992 exert their insecticidal effect by binding to the 20-hydroxyecdysone binding site and activating the ecdysteroid receptors permanently (Wing 1988; Wing et al. 1988; Smagghe et al. 1994; Wurtz et al. 2000). Their comprehensive effects and high selectivity as well as lower toxicity to non-target animals and the environment provide new tools for integrated pest management.

Oxadiazolyl 3(2H)-pyridazinone, 2-*tert*-butyl-4-chloro-5-[5′-(4′-chlorophenyl)-2′-(1′,3′,4′-oxadiazolyl)methoxy]-3(2*H*)-pyridazinone (ODP), was reported as a new growth inhibitor that possessed considerable inhibitory activity on weight gain of lepidopterans such as *Ostrinia furnacalis, Plutella xylostella, Pieris rapae, Bombyx mori* and *Pseudaletia separata* (Huang et al. 2003; Qian et al. 2005). Further reports about ODP were mainly focused on a derivatizing series of novel compounds to find satisfactory structure—activity relationships with the aim of designing more potent analogues (Cao et al. 2003, 2005). No detailed study had been done to evaluate its insecticidal properties and determine its mode of action.

The present study aimed to investigate the effect of ODP on the process of growth of larvae of the armyworm, *P. separata* Walker (Lepidoptera: Noctuidae), by feeding with drug-coated maize leaves at different stages in comparison with toosendanin, a tetranortriterpenoid extracted from the bark of *Melia toosendan* with multiple modes of action on insects as a feeding deterrent and an insect-growth regulator that disrupts the molting process. In mammals it acts as a pre-synaptic blocker at the neuromuscular junction (Schoonhoven et al. 1994; Wu et al. 2001; Cui et al. 2002). Further objectives were to determine the digestive physiological effects of ODP on the insect.

**Figure 1.**
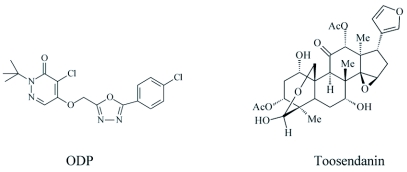

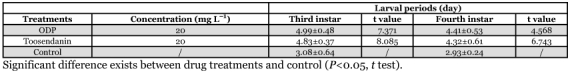
Chemical structures of oxadiazolyl 3(2H)-pyridazinone (ODP) and toosendanin.

## Materials and methods

### Chemicals

Oxadiazolyl 3(2H)-pyridazinone (93% purity) was confirmed by using ^1^H-NMR, IR, GS-MS and elemental analysis. Toosendanin (85% purity) was obtained from Shanghai Pesticide Institute, China. All the compounds used for bioassays were dissolved in N, N-dimethylformamide (DMF) and stored in a freezer (-20^°^C) until used. The stock solutions in DMF were diluted to the required concentrations with water containing Triton X-100 (0.2ml liter^-1^). The structures of ODP and toosendanin are shown in Figure 1.

### Insects

The armyworm larvae, *Pseudaletia separata* Walker (Lepidoptera: Noctuidae), were from our own laboratory culture. They were kept at 23 ± 1^°^C, 70∼80% RH under long day, 16:8 LD conditions, and were fed freshly cut maize seedling. The larvae used in the bioassays were starved for 4 hours before the experiments.

### Bioassays protocol

Bioassays were performed by feeding insects leaves dipped in the test compounds (Isman 1991). Fresh maize seedling leaves were coated with different concentration of test chemicals, air dried, and fed to the larvae. Control leaves were treated with dimethylformamide and water containing Triton X-100 (0.2 ml liter^-1^) and air dried. The experimental conditions were the same as the rearing conditions.

### Assays of larval growth

Third instar larvae and sixth-instar larvae were used for experimental treatments to test the effect of ODP on the process of larval growth. Third instar larvae were fed with the drug-coated leaves for 96 hours, the treated leaves were then removed and replaced with fresh untreated leaves until the larvae pupated. The larval period and the profiles of larval growth such as prepupa, pupa, adult eclosion and subsequent oviposition were counted. Sixth-instar larvae, however, were fed only with the coated leaves, the weight gains were recorded every 12 hours until the larvae pupated. Both of the assays were repeated five times for each concentration with a minimum of 20 larvae per replicate.

### Midgut enzyme preparations

Midguts dissected from fifth-instar larvae were homogenized in iced normal saline solution containing 20 m*M* NaCl and 0.1m*M* CaCl_2_ (50 µl/midgut), and centrifuged at 10,000 × g, 4^°^C for 20 min. The supernatant was filtered through a 0.2 µ nitrocellulose membrane filter to remove small suspended particles and bacteria, and used for studies of enzymes (Bergmeyer 1984; Li et al. 1997).

### Assays of gross protease activity

Gross protease activity was measured as described by Bergmeyer (1984). The 3.0 ml reaction mixture consisted of 1.0 ml enzyme sample and 2.0 ml 0.2 *M* phosphate buffer (pH 8.0) containing 0.5% (g/v) casein. After incubation at 37^°^C for 15 min, 3.0 ml trichloroacetic acid (20% g/v) were added to stop the reaction. The solution was centrifuged at 4,000 × g for 10 min and 1 ml of the supernatant was removed and mixed with 5.0 ml 0.55 *M* sodium carbonate solution. An analogously treated sample without casein was used as control. The colour development as a result of tyrosine formation was measured at 680 nm. The enzyme activity was calculated from a tyrosine standard curve.

### Assays of trypsine-like enzyme activity

For weak alkaline trypsine-like enzyme and chymotrypsin-like enzyme assays, the activities were measured using methods described by Campbell et al. (1987). Tosyl-L-arginnine methyl ester and benzoyl-L-tyrosine ethyl ester were used as substrates for their activities, respectively. The activity of both enzymes was determined by changes in absorbance at 247 nm from beginning to end of the reaction.

### Assays of alpha amylase activity

Alpha amylase activity was measured with 3,5-dinitrosalicylic acid using soluble starch as substrate (Nagaraju et al. 1995; Ishimoto 1999). 0.5 ml of enzyme sample was preincubated at 37^°^C for 15 min prior to the addition of 0.5 ml 20m*M* PBS (pH 5.8) buffer containing 1% soluble starch, 20 m*M* NaCl and 0.1 m*M* CaCl_2_. After incubation for 3 min, the reaction was terminated by the addition of 3.0 ml 3,5-dinitrosalicylic acid reagent (1.0% g/v), followed by boiling for 5 min in a water bath. After addition of 6.0 ml water, the solution was mixed and allowed to stand at room temperature for 15 min. Absorbance was measured at 520 nm. Maltose hydrate was used as standard.

### Protein determination

Protein content was determined by the Bradford method using bovine serum albumin as the standard (Bradford 1976).

### Data analysis

All data were subjected to analysis of variance (ANOVA) and means separated using the Tukey-Kramer statistical test. The level of significance (*P*) was set at 5% (SAS Institute 1988).

## Results and Discussion

### Effects on larval period

This study showed that the incorporation of ODP into the diet had a significant effect on both growth inhibition and direct toxic effects after ingestion. The median effective concentration (EC_50_) of ODP with a negative effect on the growth of the third instar larvae of *P*. *separata* was 36.67 ± 3.81 mg litre^-1^. A sublethal dose of 20 mg litre^-1^ ODP caused slower development and a prolonged larval period, smaller body size and sluggish behavior (Table 1). The length of larval periods of *P*. *separata* fed with drug-coated maize leaves showed that the effects of ODP were comparable with the results of toosendanin treatments.

**Table 1.**
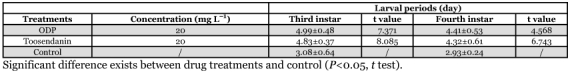
Larval periods of *P. separata* larvae fed with drug-coated maize leaves.

**Table 2.**
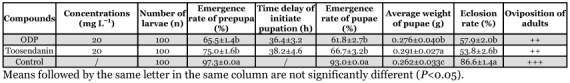
Growth development of the continuous incubated larvae after feeding with drug-coated maize leaves for 96 h

**Table 3.**
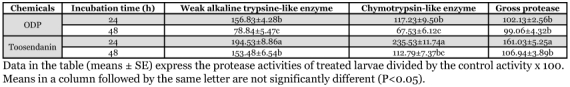
Protease activity in the midgut of fifth instar *P. separata* larvae after feeding with 40 mg litre^-1^ drug-coated maize leaves.

**Table 4.**
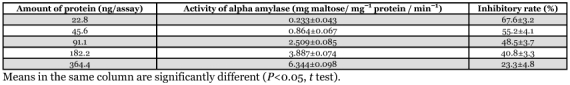
Percentage inhibition of 40 mg litre^-1^ ODP on the activities of alpha amylase from midgut of fifth instar *P. separata* larvae *in vitro.*

### Effect on growth and development by discontinuous ODP-feeding

Third instar larvae populations that were fed with fresh untreated maize leaves after an initial 96 hours exposure to treated leaves also were sensitive to both ODP and toosendanin treatments. These populations showed delayed pupation and a reduced eclosion rate of pupae and adults. Moreover, the treatments slightly decreased the fecundity of *P. separata.* But the weight of pupae in both treated groups was obviously higher than that of the control (*P*<0.05). This may be attributed to a decline of the compound's anti-feeding effects (Table 2). Furthermore, effects of both treatments showed no obvious difference on the longevity of adults and the hatching rate of eggs as compared to the control (*P*<0.05). The data indicated that the regulatory action of ODP on larval growth development was similar to that of toosendanin. Both ODP and toosendanin decreased the increasing rate of the experimental populations.

### Effect on growth and development by continuous ODP-feeding

**Figure 2.**
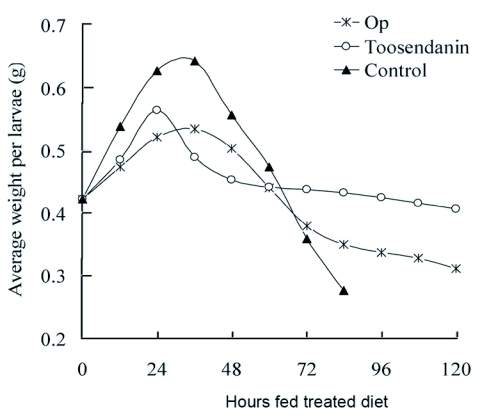
Weight gain of sixth instar *P. separata* larvae after continuous feeding with 40 mg L^-1^ drug coated maize leaves until pupation.

After rearing on the 40 mg litre^-1^ drug-coated maize leaves, weight of sixth instar larvae increased with feeding time in both drug treatments and the control (Figure 2). The maximum inhibition by ODP reached a maximum of 23.0 ± 1.8 percent at 36 hours (*P*<0.05). Pupation of ODP-treated larvae was delayed about 36.8 ± 4.3 hours relative to the control (*P*<0.05), but eclosion of prepupae was not affected. Toosendanin also inhibited the larvae weight gain, whereas 40 mg litre^-1^ toosendanin treatments promoted emergence of prepupa about 12 hours ahead of the control, and even kept the larvae permanently in the larval stage instead of pupating. It is known that the development effect of toosendanin could modify haemolymph ecdysteroid titres in larvae pupal stage in the same manner as azadirachtin (Mordue et al. 1998; Yang et al. 2002). This suggests that ODP might impair the larval growth by interfering with the endocrine system in insects.

### Effect on larval midgut protease activity

The mechanism of growth inhibition in insects given inhibitors in their diet may not always be due to the reduction of enzyme activities in the gut (Slansky et al. 1985). However, exposure to ODP in larval diets affected protease activity in the midgut of fifth instar larvae (Table 3). 40 mg litre^-1^ ODP caused a statistically significant activation of weak alkaline trypsine-like enzyme and chymotrypsin-like enzyme activity at 24 hours, but gradually became an inhibitor at 48 hours. No statistically significant differences in activities of gross protease were observed as compared to the controls (*P*<0.05). The results suggested that ODP could regulate the protein degradation in the midgut and thereby cause reduced nutrient intake that could inhibit larval growth. This reduced efficiency of utilization of digested food, and perhaps the shortage of essential amino acids, may lead to increased costs associated with growth (Boardway et al. 1986; Timmins et al. 1992). Toosendanin also enhanced the larval protease activity, and its effect gradually decreased with the feeding time as well. However, it did not change to inhibition of the larval proteases activity.

### Effect on larval midgut alpha amylase activity

The effect of 40 mg litre^-1^ ODP on midgut alpha amylase activity was measured *in vitro.* The percent inhibition by ODP was linear and negatively related to the amount of protein (Table 4).

**Figure 3.**
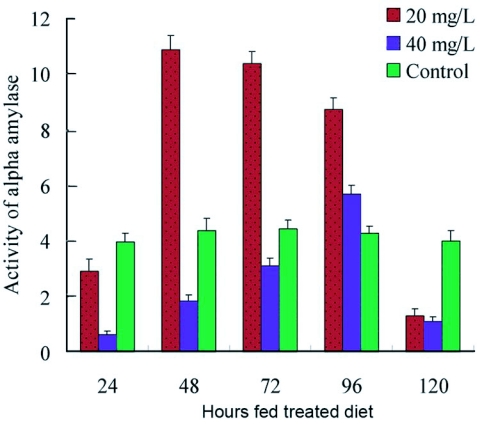
Activity *in vivo* of digestive alpha amylase of fifth instar *P. separata* larvae continuously reared with ODP-coated maize leaves.

ODP also had significant effects on alpha amylase activity of *P. separata* larvae *in vivo.* As shown in Figure 3, when fifth instar larvae were reared with 20 mg litre^-1^ ODP-coated leaves, the activity of alpha amylase was inhibited at 24 hours, but by 48 hours was activated about two times compared to the control. Activation was gradually decreased with feeding time until the inhibition was restored at 120 hours. At the higher dose of 40 mg litre^-1^ ODP alpha amylase activity was more slowly activated. These results suggest that the effect of ODP on alpha amylase was dependent on drug dose. The reduction of the activity of alpha amylase activity in the midgut would be expected to have severe consequences on carbohydrate assimilation and the efficiency of food digestion by larvae.

In summary, oxadiazolyl 3(2H)-pyridazinone, a potent effective IGR, regulated the process of growth of *P. separata* larvae. The mode of action might have comprehensive effects on the rate of growth of insect populations. The anti-feeding effect of oxadiazolyl 3(2H)-pyridazinone might be attributed to its interfering action on many kinds of important enzymes such as weak alkaline trypsine-like enzyme, chymotrypsin-like enzyme, and alpha amylase in the guts of insects. However, the observed disruption of larval growth physiology could also be caused by an effect on gustatory receptors. Since inhibitors of digestive enzymes could be used to protect plants against insects, oxadiazolyl 3(2H)-pyridazinone could be a pro-insecticide for the development of novel pesticides to satisfy the practice of agricultural pest insect control.

## Abbreviations

ODP -: oxadiazolyl 3(2H)-pyridazinone
